# Imaging Genomics Across Primate Species: Advancing Understanding of Neurological and Psychiatric Disorders

**DOI:** 10.1007/s12264-026-01627-0

**Published:** 2026-04-28

**Authors:** Tingting Bo, Yichun Huang, Yong Lu, Yang Song, Jiangtao Zhang, Jianmin Zhang, Heqiu Wang, Zheng Wang

**Affiliations:** 1 Department of Endocrine and Metabolic Diseases, Shanghai Institute of Endocrine and Metabolic Diseases, Ruijin Hospital, Shanghai Jiao Tong University School of Medicine, Shanghai, 200025 China; 2 Department of Radiology, Ruijin Hospital, Shanghai Jiao Tong University School of Medicine, Shanghai, 200025 China; 3 Clinical Neuroscience Center, Ruijin Hospital Luwan Branch, Shanghai Jiao Tong University School of Medicine, Shanghai, 200020 China; 4 School of Psychological and Cognitive Sciences; Beijing Key Laboratory of Behavior and Mental Health; National Key Laboratory of General Artificial Intelligence; IDG/McGovern Institute for Brain Research; Peking-Tsinghua Center for Life Sciences, Peking University, Beijing, 100871 China; 5 Department of Radiology, Central Hospital of Dalian University of Technology, Dalian, 116033 China; 6 Tongde Hospital of Zhejiang Province (Zhejiang Mental Health Center), Zhejiang Office of Mental Health, Hangzhou, 310012 China; 7 School of Biomedical Engineering, Hainan University, Haikou, 570228 Hainan China

**Keywords:** MRI-derived phenotypes, Psychiatric diseases, Transcriptome analyses, Non-human primates

## Abstract

This review delves into brain imaging genomics, an interdisciplinary field merging brain imaging, genomics, and additional biomarkers with clinical data. The primary aim is to uncover new insights into the brain’s phenotypic, genetic, and molecular characteristics. We emphasize recent advances in genome-wide association studies and transcriptome-wide association studies, especially their integration with MRI-derived phenotypes in humans. These studies are crucial for understanding how various factors influence brain structure and function in normal and pathological states. Furthermore, this review highlights imaging transcriptomics progress in non-human primates, essential for elucidating brain organization and improving animal models evolutionarily to bridge gaps in understanding human disorders. We conclude that brain imaging genomics is set to transform research in neurological and psychiatric disorders, owing to its holistic approach that merges advanced genetic analysis with detailed imaging, will deepen our understanding of the brain, and usher in a new epoch in brain imaging research.

## Introduction

Human behaviors emerge from intricate interactions across biological systems, spanning from genes and proteins to cells and biological pathways. To comprehend how the neurobiological process correlates with specific behaviors, a multiscale approach is essential. This encompasses assessments of brain structure and function, alongside genetic and transcriptional data. Brain imaging genomics represents a cutting-edge intersection of various scientific domains, encompassing brain imaging, genomics, and other biomarkers, which is integrated with clinical and behavioral data, aiming to unravel the intricate phenotypic, genetic, and molecular characteristics of the brain. This field’s emergent nature holds significant promise for expanding our understanding of the brain’s functionality, both in health and disease. Brain imaging genomics typically involves the concurrent analysis of imaging data with genome-wide association studies (GWAS) and transcriptome-wide association studies (TWAS). GWAS facilitates the integration of imaging data with a spectrum of genetic variations, including single-nucleotide polymorphisms (SNPs), epigenetic modifications, copy number variations (CNVs), and molecular characteristics identified through various omics methodologies [1]. Gene enrichment analyses from GWAS have indicated that certain cortical regions may be more susceptible to diverse factors that elevate the risk of brain disorders [2]. Conversely, TWAS amalgamates imaging data with brain bulk tissue transcriptome or single-cell/nucleus transcriptome analysis.

The brain exhibits intricate gene expression patterns, characterized by a multitude of expressed genes, including noncoding RNAs, and a plethora of alternative splice isoforms [3]. Psychiatric traits, inherently linked to the human brain’s vast cellular diversity and connectivity, are fundamental to neural functioning [4]. The convergence of advanced genetic analysis and detailed brain imaging inherent in brain imaging genomics is revolutionizing research in neurological and psychiatric disorders. This holistic approach is instrumental in deepening our understanding of the brain, potentially leading to novel diagnostic and therapeutic strategies. Imaging transcriptomics in non-human primates is a critical aspect of brain imaging genomics. These studies are indispensable for comprehending brain organization and development from an evolutionary perspective. They play a crucial role in refining animal models, thus bridging the gap in our understanding of human brain organization and related disorders. This review aims to present recent advancements in human brain imaging genomics and highlight the significance and potential of nonhuman primate transcriptomes in future interspecies exploration.

## Multiscale Insights into Brain Structure, Function, and Pathology

Whole-genome transcription analyses have elucidated genetic variants linked to symptoms of various human mental illnesses, including major depressive disorder (MDD) [5, 6], bipolar disorder [7], schizophrenia [8, 9], Alzheimer’s disease (AD) [10–13], and Parkinson’s disease (PD) [14, 15]. Neuropsychiatric disorders are predominantly polygenic, with potentially hundreds or thousands of contributing genetic variants dispersed across the genome. Importantly, no single variant fully explains the etiology of these diseases [16, 17]. Moreover, expanding our focus to the transcriptome level provides insight into the molecular mechanisms underpinning complex genetic risks in these disorders. Variations in genes, coupled with epigenetic changes, can influence transcript levels in a dose-dependent manner or through transcription factors. The transcriptional process, following the central dogma, acts as a pivotal link between genetic polymorphisms and intermediate phenotypes, thus offering a crucial genetic foundation for understanding disease and cognition. Advanced technologies such as single-cell and bulk tissue transcriptomics have shed light on the molecular diversity within specific brain regions and their alterations in psychiatric diseases [18]. For instance, single-nucleus transcriptomics has revealed distinct gene expression patterns across brain regions in various diseases, including MDD [19], autism spectrum disorder (ASD) [20, 21], and AD [10]. Human bulk tissue transcriptomics has further shown that these disorders may exhibit shared or unique spatial and temporal characteristics. Notably, a single-cell atlas of the human substantia nigra has uncovered that different neuropsychiatric disorders (AD and PD) converge on shared loci but are associated with unique neuron-specific genes [22]. Additionally, TWAS and mRNA expression analyses in anorexia nervosa (AN) and obsessive-compulsive disorder (OCD) have identified common association signals in distinct brain regions and developmental stages [23].

A promising approach to advancing our understanding of genetic risk in psychopathology involves identifying convergent disease-related genes and pathways that characterize common loci, shared genetic structures, and specific behaviors across different disorders [24]. Genome-wide analyses have categorized eight psychiatric disorders into three interconnected groups [25]. The first group encompasses disorders characterized by compulsive behaviors, including AN, OCD, and Tourette syndrome. The second group comprises mood and psychotic disorders such as MDD, bipolar disorder, and schizophrenia. The third group includes disorders related to early-onset neurodevelopment, namely ASD and attention-deficit/hyperactivity disorder (ADHD), and represents a convergence of elements from the first two groups. Moreover, GWAS meta-analysis of six large depression datasets revealed that depression is highly polygenic, with ~11,700 variants, estimating that >95% of risk variants for other psychiatric disorders (anxiety, schizophrenia, bipolar disorder, and ADHD) were influencing depression risk when both concordant and discordant variants were considered [26].

## Unraveling the Impact of Genetic Variants on Imaging Traits

The robust association of hundreds of genetic variants with psychiatric disorders has been well-documented in extensive human genetic datasets. However, the efficacy of transcriptomic approaches in uncovering mechanisms underlying the genetic risk for these disorders, particularly given their polygenic nature, remains a subject of ongoing research. Current strategies, including brain imaging phenotyping combined with genetics, are shedding new light on brain genetics. Makowski and coworkers utilized genetically informed brain atlases to demonstrate that brain hierarchy and morphogenetic gradients of cortical surface area and thickness correlate with a genetic landscape encompassing both conserved and human-specific features. These features, identifiable in adulthood, are linked to early neurodevelopment and neuropsychiatric risk pathways [27]. Similarly, van der Meer *et al.* observed genetic overlaps between total surface area and mean thickness, noting that regional surface area is more discoverable and less polygenic than regional thickness measures [28]. Moreover, shared genetic underpinnings for cortical brain structure and blood immune markers were found in the UK Biobank dataset, with implications for neurodevelopment and understanding the etiology of brain-related disorders [29]. Genetic architecture analysis of fornix white matter microstructure revealed that 63 independent significant variants within 20 genomic loci were associated with the six fornix diffusion MRI traits [30]. In schizophrenia research, GWAS analyses of structural MRI metrics identified three genomic regions, on chromosomes 3p21, 17q21, and 11p11, enriched for neurodevelopmental processes and implicated in associations between schizophrenia and cortical network organization [31]. Beyond the cerebral cortex, the cerebellum’s role in cognitive and emotional functions, and its association with various psychopathologies, is gaining recognition. Chambers *et al.* investigated the common allele architecture of cerebellar volume, discovering genetic correlations with other brain volumes and psychiatric phenotypes, including schizophrenia, bipolar disorder, and ASD [32].

Functional magnetic resonance imaging (fMRI) has revealed abnormal activations in multiple brain networks in neurological and psychiatric patients, such as the default mode, central executive, attention, limbic, salience, somatomotor, and visual networks [33, 34]. A comprehensive understanding of these networks necessitates identifying the common and rare genetic risks associated with complex traits. Moreau *et al.* demonstrated that heterogeneity and polygenicity affect the detection of brain connectivity abnormalities underlying psychiatric symptoms [35]. A recent study combining GWAS with independent-component analysis of fMRI data validated 45 novel genetic regions associated with a triple-network model of psychopathology, including central executive, default mode, and salience networks. These variations in brain function were found to be genetically correlated with brain disorders such as MDD and schizophrenia [36]. Further research linking GWAS with both functional and structural brain imaging phenotypes indicates that the genetic architecture of the brain is relevant to both brain development and aging [37, 38]. Three brain age gaps derived from gray matter volume, white matter microstructure, and functional connectivity were identified, linked to sixteen genomic loci, and the gray matter volume-brain age map displayed the most pronounced heritability enrichment in genetic variants within conserved regions [39].

Efforts to understand the relationship between imaging measurements and single gene expression have also been noteworthy [40]. A systematic review focusing on resting-state functional connectivity and genes associated with AD reported consistent associations with the APOE-ε4 allele and deficits in the default mode network [41]. Specifically, blood transcription and proteomics seem to be more feasible in future human research. Large-scale plasma proteomics data have highlighted potential regulatory proteins and pathways for diagnostic and therapeutic strategies in neurodegenerative diseases [42]. Ossenkoppele *et al.* demonstrated that age, Aβ status, APOE ε4 carriership, and female sex were all associated with a higher prevalence of tau positron emission tomography (PET) positivity and further put forward the direction of comparing the tau PET prevalence estimates against plasma markers of soluble tau pathology, such as p-tau217 [43, 44]. A study combining PET, cerebrospinal fluid, and plasma biomarkers to detect AD pathology revealed that plasma p-tau181 could serve as a candidate predictive biomarker in the late clinical stage of AD [45]. Moreover, in women with AD, the plasma beta-amyloid 42/40 ratio was a potential biomarker for brain metabolism, and imaging indicators displayed consistent correlation curves with progression [46]. Collectively, these results demonstrate a coordinated interplay between peripheral blood proteins and the central brain transcriptome throughout the course of AD. Muehlhan and colleagues also found an association between blood-derived SLC6A4 promoter methylation and resting-state functional coupling between the amygdala and salience network regions [47], while Ismaylova *et al.* reported a stronger association with frontal-limbic resting-state functional connectivity [48]. In autism, multivariate fMRI responses have been linked to blood leukocyte transcriptomes enriched in ASD-associated, prenatal, human-specific, and language-relevant genes [49].

The integration of advanced imaging phenotypes is also facilitating the discovery of non-invasive markers for brain health. For example, quantitative susceptibility mapping (QSM), an MRI technique measuring tissue magnetic susceptibility, has been effective in detecting pathological changes in tissue iron, myelin, and calcification. Elliott *et al.* established a link between iron transport and storage genes and subcortical brain susceptibility [37]. Furthermore, genetic and phenotypic associations of QSM in the human brain revealed 76 replicating clusters of genetic variants and 251 phenotypes, including body iron, disease, diet, and alcohol consumption, uniquely related to magnetic susceptibility [50]. Research integrating QSM, pathological data from post-mortem AD brain, along with transcriptomic data from the AHBA, even provided quantitative insights into the variable vulnerability of cortical regions to higher levels of Aβ aggregation, iron overload, and subsequent neurodegeneration in AD, indicating changes preceding clinical symptoms [13].

Collectively, these efforts are crucial in delineating the genetic structure of normal brain development and diseased brains. The current progress in linking genetic risk variants to imaging phenotypes may provide a roadmap for enhancing future GWAS and imaging association studies. Identifying the most discoverable measures is vital for advancing our understanding of brain morphology, function, and clinical disorders.

## Linking Gene Expression Profiles to Brain Imaging Phenotypes

**Fig. 1:**
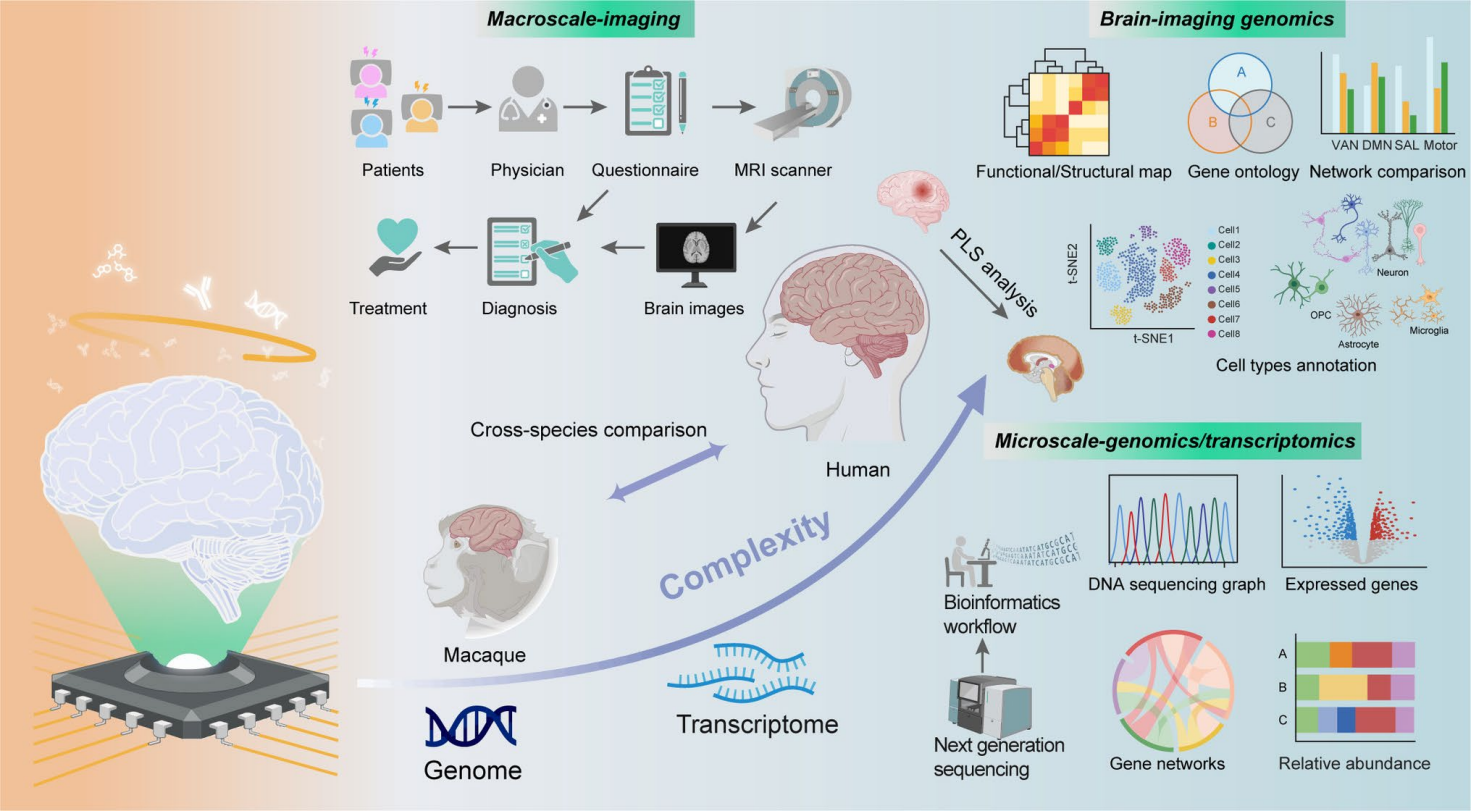
Conceptual Overview. The convergence of large-scale imaging datasets and transcriptomics has unlocked new opportunities for exploring the underlying mechanisms of imaging-derived phenotypes. On one hand, extensive brain imaging data from diverse patients, accompanied by clinical assessments and behavioral characterizations, have been leveraged for disease diagnosis, stratification, and treatment. On the other hand, various biochemical and genetic measurements from brain tissue and blood have elucidated causes of disease onset and progression from different perspectives. Recent integrative analyses combining imaging with transcriptomic and genomic changes have advanced mechanistic insights into the macroscopic properties of brain organization, enabling elucidation of the mechanisms underlying brain disorders, as well as cross-species brain activity patterns and cortical morphological changes at a genetic level.

The exploration of molecular mechanisms underlying common imaging-derived phenotypes has gained momentum with the amalgamation of large-scale imaging datasets and transcriptomics (Fig. 1). This synergy has facilitated mechanistic insights into the macro-scale properties of brain organization, significantly influencing spontaneous brain activity patterns and cortical morphology. Several extensive repositories, including the Allen Human Brain Atlas (AHBA) [51, 52], UK Biobank [53], and Alzheimer’s Disease Neuroimaging Initiative (ADNI) [54], have provided new perspectives on how spatial molecular variations correlate with macroscopic neuroimaging phenotypes. In addition, transcriptome atlases focusing on specific developmental periods or regions offer invaluable, freely accessible resources for targeted inquiries into brain development [55–58]. Established pipelines now facilitate the integration of brain-wide transcriptomic and neuroimaging data, and the impact of different processing choices on the resulting data has been comprehensively evaluated [59]. Prior to these public datasets, a comprehensive understanding of the evolution, formation, and pathological disruptions of human brain circuits was hindered by data limitations.

Utilizing the AHBA gene expression data, a significant correlation has been identified between 136 genes and resting-state fMRI activities across the dorsal default mode, salience, visuospatial, and sensorimotor networks, as reported by Richiardi *et al.* [60]. This research also confirmed a link between conserved gene expression and functionally relevant brain circuitry, a finding echoed by Hawrylycz *et al.* [61]. In a focused study of 10 human neocortical areas, Wang *et al.* discovered specific genes correlated with resting-state activities in the default mode network, predominantly enriched in neuronal cells [62]. Furthermore, extensive molecular research has provided insights into various facets of fMRI, including inter-modular hubs and connection distances [63], functional connectivity dynamics [64], functional organization across corticocortical and cortico-striatal circuitry [65, 66], as well as aging-related changes in resting-state brain networks [41].

MRI studies have also demonstrated correlations between brain structural changes during development and transcriptional profiles, impacting cortical thickness, gray matter volume, and magnetization transfer [67–73]. Meanwhile, three patterns of gene expression have also been found to reveal convergent links between healthy brain organization and neurodevelopmental disorders. One was associated with functional connectivity strength, another with theta oscillations, and the third with adolescent change in gray-matter myelination [74]. Additionally, Liu and coworkers have revealed sex-specific gene signatures that are significantly associated with gray matter volume variations, linked to the regional expression of sex-chromosome genes, and characterized by unique cell-type signatures in adult humans [75].

To be mentioned, the AHBA dataset was derived from a limited number of individuals, which may introduce several key limitations. Firstly, individual variability poses the most direct and severe impact, as gene expression variations influenced by age, sex, genetic background, lifestyle, and even post-mortem interval may be disproportionately driven by the unique characteristics of just six donors. Secondly, low statistical power resulting from a small sample size forces stringent multiple comparison corrections, potentially filtering out genuine but weak gene-phenotype associations. Thirdly, potential limited generalizability arises from the homogeneous sample composition (predominantly European adults without neurological disorders), restricting population-level inferences. Fourthly, spatial alignment challenges emerge when standardizing heterogeneous gene expression data to standard brain atlases (e.g., MNI space), introducing cross-modal comparison errors.

Despite these limitations, the AHBA remains an indispensable resource, and various ways can be developed to improve reproducibility and solve the heterogeneity: (1) Rigorous data screening and quality control [76]. When using the AHBA, genes with extremely low expression levels or those undetected in most samples are typically filtered out. Corrections can also be applied for factors such as RNA quality (RNA integrity number value); (2) Utilizing "consensus" gene expression maps. Instead of analyzing data from each donor individually, a "group-average" gene expression map across donors is generated through averaging or other statistical methods to smooth out extreme individual differences, which is commonly used in TWAS and translational studies [77, 78]; (3) Principal component analysis and confounding factor correction. Using gene expression profiles of the first one or two components to control for unknown batch effects and inter-individual variations [68, 79]; (4) Enrichment analysis over single-gene analysis. The analytical focus shifts from "individual genes" to "gene sets" or "pathways" [80–83]. For example, examining whether group-level expression patterns associated with specific cell types (e.g., oligodendrocytes) or specific biological functions (e.g., synaptic transmission) correlate with imaging phenotypes; (5) Independent validation, which is crucial. Any findings based on the AHBA should be regarded as "hypothesis-generating" rather than "conclusive verification." It is essential to seek validation in independent samples (tissue data from another cohort, such as the PsychENCODE Consortium) [84–86]; (6) Leveraging complementary data sources. Integrating inferred gene expression from genotypic data (e.g., through TWAS or GWAS) to cross-validate discoveries [87, 88]. These methods utilize the statistical power of large GWAS samples and can serve as strong supplements to findings from the AHBA.

## Associations between Transcriptomics, Imaging Phenotypes, and Behavioral Symptoms in Disease

The limited availability of patient-derived tissue samples amplifies the importance of analyzing transcriptional patterns associated with distinct imaging features in psychiatric disorders. Such analysis is integral for comprehending the anomalies in structure and function at a molecular level. It facilitates the understanding of how transcriptomic outcomes connect molecular mechanisms with the broader framework of connectome organization, thus unraveling the molecular pathology in various psychiatric disorders [70, 89]. Genomic research and imaging studies have made strides in identifying shared genetic and neural circuit disruptions across multiple psychiatric conditions [3, 90, 91]. The comparison of differential gene expression against imaging phenotypes is key to understanding the molecular pathology at the level of the brain [92]. Additionally, the use of multivariate techniques, particularly partial least squares regression, is well-suited for integrating complex datasets like neuroimaging and genetic information. This approach is critical for investigating the association between brain abnormalities and transcriptional activities in psychiatric disorder cases [93].

*Major Depressive Disorder*

Major Depressive Disorder (MDD) is a multifaceted disorder arising from the intricate interplay of biological systems, spanning the molecular to the behavioral spectrum. Imaging studies have revealed that MDD is characterized by structural and functional anomalies in key brain regions involved in emotional and cognitive functions, including the frontal cortex, parietal cortex, thalamus, and caudate [34, 94–96]. Distinctive dynamic connectivity patterns linked to long-term depression genes have been observed [97]. An integrated approach, combining imaging and transcriptomic data, can bridge the gap between genes, cellular classes, and biological pathways, and the *in vivo* imaging manifestations of depression. Studies that have merged brain-wide gene expression analyses with morphometric changes in MDD patients demonstrate a spatial correlation between the expression of MDD-related genes and structural variances detected by morphometric similarity networks (MSNs) [98]. Notably, transcriptional alterations in microglia and neurons largely explain the observed correlation with MDD-specific MSN differences [80]. Further integration of diverse data sets, including imaging, cortical gene expression, single-cell gene expression, postmortem patient transcriptional data, and depression GWAS, has revealed converging molecular, cellular, and cortical neuroimaging signatures of MDD. This includes the identification of somatostatin interneurons and astrocytes as consistently associated cells in depression, corroborating the enrichment of GWAS-derived polygenic risk for depression in genes expressed in interneurons [99]. Moreover, transcriptional correlations with depression imaging phenotypes have been linked to gene downregulation in postmortem cortical samples from patients with depression. Additionally, sex-specific regional gene expression signatures corresponding to functional connectivity changes in depression have been identified, which may pave the way for new biomarkers and fMRI-guided therapeutic neuromodulation strategies [100].

*Autism Spectrum Disorder*

Autism Spectrum Disorder (ASD) is a diverse neurodevelopmental disorder characterized by challenges in social cognition, self-representation, language, and face processing. These challenges have been linked to atypical activities in the ventromedial prefrontal cortex, thalamus, visual areas, amygdala, and posterior cingulate [101–103]. The correlation between fMRI findings and gene expression levels in blood leukocytes provides a vital *in vivo* perspective for unraveling brain-relevant molecular mechanisms in ASD [49]. Understanding the multi-level heterogeneity between fMRI findings and brain transcriptomics in ASD is crucial for both clinical and translational research. Studies have identified transcriptional variances in specific brain regions of individuals with ASD [104, 105]. Notably, RNA-sequencing analysis conducted on 11 cortical areas from post-mortem ASD brain samples revealed extensive transcriptomic alterations across the cortex, with a noted reduction in transcriptomic diversity between cortical regions [106]. The autism brain imaging data exchange (ABIDE I and II) has enabled focused research on molecular and network-level mechanisms underlying individual differences in ASD [107, 108]. Combining subtyping of functional brain connectivity patterns and the Allen transcriptomic data characterized a link between excitation/inhibition imbalance and functional connectivity alterations, but only in one ASD subtype, overall characterized by brain hyperconnectivity and major alterations in somatomotor and default mode networks [109]. A subset of genes in individuals with ASD has been found to follow a divergent developmental trajectory, and these genes are predominantly involved in voltage-gated ion channels and inhibitory neurons, indicating a potential excitation-inhibition imbalance in ASD [110]. Furthermore, combining neuroimaging with gene expression data has illustrated that functional connectivity differences in ASD subgroups can be attributed to regional variations in the expression of specific ASD-related gene sets. These genes are linked to distinct molecular signaling pathways, including immune and synaptic functions, G-protein-coupled receptor signaling, protein synthesis, and more [111]. Through an integrated analysis of genome-wide screening, single-cell sequencing, and brain imaging data, several brain regions were identified contributing to ASD etiology, such as the precentral gyrus, postcentral gyrus, superior temporal region, and sensory areas [112]. Additionally, transcriptomic variation and cortical morphology differences analyses have shown that synaptic and transcriptionally downregulated genes significantly contribute to variations in global cortical thickness in children with autism [113].

*Schizophrenia*

Despite significant advancements in neuroscientific research, the biological mechanisms underlying psychotic disorders remain elusive. Extensive studies have been conducted to explore both the architectural and functional changes in the brain associated with schizophrenia [114–118]. Efforts have been made to unveil promising neuroimaging biomarkers for schizophrenia, and polygenic genetic variants were assessed to estimate the likelihood of an individual developing complex diseases [119]. A global pattern of accelerated cortical thinning in schizophrenia has been identified, and genes down-regulated in cortical regions that exhibit this accelerated thinning were expressed at lower levels in several psychiatric disorders and were enriched for both common and rare disrupting variation for schizophrenia and neurodevelopmental disorders [120]. A notable aspect of recent research has been the use of intracortical magnetization techniques, as evidenced by microstructural MRI maps. These advanced imaging modalities have been instrumental in linking observable brain changes with schizophrenia-like traits. Romero-Garcia *et al.* provided significant evidence in this domain, correlating these imaging findings with previous histological data on dysregulated gene expression in schizophrenia. A groundbreaking study by Li *et al.* identified a neuroimaging biomarker for schizophrenia, marking a significant step in psychiatric diagnostics. This biomarker, characterized by functional striatal abnormalities, was found to be spatially correlated with the dopaminergic function. Moreover, these abnormalities were connected to the expression profiles of schizophrenia risk genes, such as *DRD2* and *GRM3*. This finding, derived from a combination of fMRI analysis and the AHBA gene expression datasets, underscores the potential of neuroimaging in understanding and identifying psychiatric disorders at a molecular level [121]. Morphometric similarity analysis has also been applied to study psychosis, focusing on markers of interareal cortical connectivity in psychosis. Morgan *et al.* observed that the cortical map of differences between cases and controls was intricately associated with the brain expression of schizophrenia-related genes [79]. This connection between morphometric data and gene expression patterns provides a more comprehensive understanding of the cortical alterations in schizophrenia. Anderson *et al.* made an intriguing discovery linking interneuron-related transcripts with individual differences in schizophrenia risk [122]. Their research suggests that these transcripts can significantly influence the molecular-genetic basis of brain function in the general population. This finding points to the intricate relationship between specific cell types, such as interneurons, and the broader genetic susceptibility to schizophrenia. This exploration demonstrates the increasingly sophisticated methods used in schizophrenia research, combining neuroimaging and transcriptomics. Such integrative approaches are pivotal for unraveling the complexities of schizophrenia and paving the way for precise, targeted treatments.

## Imaging Transcriptomics and Molecular Landscape of Monkey Brain

In the quest to unravel the complexities of human diseases, remarkable progress has been achieved in imaging-genetics and transcriptomics, particularly in analyzing diverse brain structural phenotypes and their links to genetic and transcriptomic disease risks in specific patient cohorts. Equally important is the understanding of the evolution and development of the human nervous system, particularly in areas like the prefrontal cortex, as highlighted in the literature [123, 124]. Non-human primates, especially macaques, have emerged as critical models for studying advanced cognitive functions due to their evolutionary, behavioral, and developmental parallels with humans [125, 126]. Genetic theory posits that morphological evolution occurs primarily through altered expression of conserved proteins, driven mainly by mutations in the cis-regulatory elements of pleiotropic developmental regulators and their downstream target genes [127, 128]. Specifically, evolutionary divergence of brain regions between species may be shaped by a combination of mechanisms, including dose effects, rewiring of gene regulatory networks, and sequence-driven neofunctionalization, such as novel genes, protein sequence divergence, and alternative splicing [129–132]. Linking gene expression differences to cognition requires converging evidence beyond statistical significance, including spatial specificity—localization to higher-order cognitive brain regions; temporal specificity—occurrence during critical developmental windows (e.g., neurogenesis); human specificity—expression patterns unique to humans and not shared with other primates; functional validation—using brain organoids or animal models to test causal effects [124, 133, 134].

Recent breakthroughs in gene editing, germline transmission, and cloning techniques have enabled the replication of human diseases in macaques, facilitating the development of innovative diagnostic and treatment technologies [135–140]. Digital macaque brain templates and atlases have become indispensable tools in characterizing specific brain structure [141–147] and function changes [138, 148, 149] using MRI, enabling cross-species comparisons [150–152].

While the transcriptomic and imaging features of primate brains have been explored separately from an evolutionary perspective, comprehensive characterization across tissues and developmental stages in macaques has been detailed [153–158]. Although limited to a few brain regions, the transcriptional architecture of the macaque brain has been validated as more akin to humans than rodents [159, 160] and applied to investigate human diseases such as MDD [161]. Studies have also reported convergent and unique transcriptional features in the brains of humans, macaques, and other animals [162–168]. MRI studies have further revealed the conservation of structure and neuronal connectivity across humans and macaques [98, 169–172], indicating that spatial patterning of gene expression and imaging measurements are closely linked in both species. Notably, human-accelerated genes show differential expression in higher-order cognitive networks in humans compared to chimpanzees and macaques [173]. MRI-derived T1-weighted/T2-weighted mapping has captured a hierarchy of transcriptomic specialization across humans, consistent with monkey microanatomy, and linked to microcircuit function and neuropsychiatric disorders [174]. Recent development of a comprehensive anatomically-defined atlas of brain transcriptomics from large-scale bulk RNA-seq data in macaque monkeys has clarified brain-wide and cell-specific transcriptomic insights into MRI-derived cortical morphology, identifying 1, 005 genes related to cortical thickness and enriched for neurons and oligodendrocytes [175]. With this dataset, 150 noncoding genes were also found to explain variations in resting-state activity, which connected to the function of nonneuronal cells such as oligodendrocytes and linked to both autism and schizophrenia risk genes [152]. Further genetic underpinnings of arousal in macaque monkeys were examined with this brain-derived transcriptome, and transcriptional insights into state-specific regulation of electrical stimulation in the intralaminar thalamus of macaque monkeys revealed that 2,489 genes were preferentially expressed within this arousal network, notably enriched in potassium channels and excitatory, parvalbumin-expressing neurons, and oligodendrocytes [176]. These progresses pave the way for further investigation of imaging transcriptomics characteristics between human and macaque brains, including functional networks and tractography, to deepen our understanding of the primate brain. It is noteworthy that specific transgenic mouse models (such as 5xFAD, which contains five familial AD mutations), when integrated with imaging and transcriptomic data, can also provide unprecedented biological explanations for abnormalities in disease-related imaging metrics, despite the substantial differences between mouse and human brains [177].

## Conclusion

In summary, the synergy of brain imaging, genetics, and transcriptomics is poised to significantly advance our understanding of the brain and identify potential therapeutic targets. Initially, this integrative approach can pinpoint both shared and unique genetic risks associated with imaging phenotypes across various psychiatric disorders. This insight may clarify the common neuropsychiatric symptomatology observed in CNVs. Subsequently, advancements in high-field MRI, sequencing, and machine learning enable detailed exploration of the associations between brain structure, function, and various cellular and genetic elements. Furthermore, combining whole-brain imaging and transcriptomics in humans and macaques can elucidate the molecular mechanisms behind consistent regional brain disruptions, aiding in the development of targeted neuromodulation techniques.

Future research may leverage human-monkey cross-species orthologous gene sets, grounded in sequence similarity, to effectively link animal model studies with human neurological disorders. This approach will involve differential and correlation analyses in human and monkey datasets, integrated with imaging data, to establish species-specific and cross-species gene-imaging correlations. Additionally, standardization and integration of expression data across species, using refined statistical methodologies, will facilitate transcriptome comparisons between humans and monkeys. These comparisons, including analyses of interspecific differences and phylogenetic relationships, will provide insights into evolutionary perspectives of species differentiation.
